# Patient’s Reported Outcome Following Chevron Distal Osteotomy Versus SERI (Simple, Effective, Rapid and Inexpensive) Procedure in Hallux Valgus Surgery

**DOI:** 10.7759/cureus.95056

**Published:** 2025-10-21

**Authors:** Ahmad Alajlan, Eyad Alahmeri, Mohammed A Alshwieer, Abdulaziz Alabbad, Bandar Alharbi, Alanood Alharthi

**Affiliations:** 1 Department of Orthopedic Surgery, Security Forces Hospital, Riyadh, SAU; 2 Department of Orthopedics, Security Forces Hospital, Riyadh, SAU; 3 Department of Orthopedic Surgery, Maternity and Children Hospital, Buraydah, SAU; 4 Department of Medicine, Princess Nourah Bint Abdulrahman University, Riyadh, SAU

**Keywords:** chevron's osteotomy, fhsq, foot health, footwear, hallux valgus, ima angle, seri procedure

## Abstract

Introduction

This study compares patient-centered outcomes following Chevron osteotomy and the minimally invasive SERI (Simple, Effective, Rapid and Inexpensive) technique for hallux valgus correction. Despite achieving radiographic success, patient dissatisfaction persists, necessitating evaluation beyond radiological parameters. Using the Foot Health Status Questionnaire (FHSQ) and pain scoring tools, this research assesses postoperative generic and foot specific quality of life to determine which surgical method provides superior results and inform evidence-based clinical practice.

Methodology

Data were retrospectively collected from patients at Security Forces Hospital, Riyadh (2022-2024). Eligible patients with isolated hallux valgus were contacted two to four years post-surgery. Outcomes were assessed via the FHSQ, administered electronically or by phone. Statistical analysis using SPSS included comparing FHSQ domain scores (foot pain, function, footwear, general foot health) between surgical groups, with subgroup analysis by deformity severity. Significance was set at p < 0.05.

Results

Of 58 eligible patients, 41 (70.7% response rate) completed the study. The SERI group (n = 24) and Chevron group (n = 17) had a mean age of 32.5 years. Analysis revealed SERI osteotomy resulted in statistically superior patient-reported outcomes in two key domains: foot function (mean = 73.96 vs. 53.56, p = 0.023) and general foot health (78.33 vs. 57.21, p = 0.008). This advantage was most pronounced in patients with moderate preoperative deformity. No significant differences were observed for foot pain or footwear comfort. The findings suggest the minimally invasive SERI technique may offer better functional recovery and perceived foot health compared to the Chevron procedure.

Conclusion

Both techniques effectively treat hallux valgus. The SERI osteotomy demonstrated superior patient-reported outcomes in function and general foot health for moderate deformities, attributed to its minimally invasive nature. However, long-term pain relief was comparable. Patient-reported outcome measures (PROMs) are crucial for evaluating success.

## Introduction

Hallux valgus, widely referred to as a bunion, is among the most frequent forefoot deformities worldwide. A systematic review published in 2023 reported a global prevalence of approximately 19%, with significantly higher rates in women (24%) compared to men (11%), and increasing prevalence with age [1]. Regarding the pathogenesis of the disease, hallux valgus arises from the progressive failure of medial joint stabilizers, leading to metatarsal head migration and sesamoid subluxation. Lateral deviation of the proximal phalanx occurs due to adductor hallucis tension, while tendon realignment exacerbates deformity. Concurrent plantar migration of the abductor hallucis reduces its corrective ability, leading to pronation and joint incongruity [2,3].

Pain in hallux valgus may arise from multiple anatomical and biomechanical factors. These include inflammation or degenerative alterations in the first metatarsophalangeal (MTP) joint, irritation from bunion pressure against footwear, and transfer metatarsalgia due to altered forefoot load distribution. Additionally, dorsiflexion of the distal phalanx may elevate and crowd the second toe, contributing further to foot discomfort, dysfunction, and ultimately gait changes [2,4,5].

More than 130 surgical procedures have been described for hallux valgus correction [6], with multiple open and minimally invasive techniques (MIS). As numerous techniques have demonstrated satisfactory outcomes, a consensus gold standard is absent, rendering the selection of a specific procedure largely surgeon-dependent. Despite the existence of over 100 described methods, the chevron osteotomy remains one of the most rigorously studied and frequently utilized open approaches [7]. The SERI (Simple, Effective, Rapid and Inexpensive) technique, introduced as a minimally invasive option, uses a percutaneous osteotomy stabilized with a Kirschner wire and is designed to minimize soft tissue trauma and facilitate faster recovery [8]. The SERI technique achieves comprehensive radiographic correction - addressing the hallux valgus angle (HVA), intermetatarsal angle (IMA), and distal metatarsal articular angle (DMAA) - while conferring distinct clinical benefits over traditional methods, including minimal soft-tissue dissection through a smaller incision, shorter operating times, less expensive fixation hardware, and no risk of subsequent pain from retained implants [9]. While both are widely practiced, direct comparisons from the perspective of long-term patient-centered outcomes as representative of open and MIS approaches remain limited.

A well-documented dissociation exists between radiographic success and patient satisfaction following hallux valgus correction [10]. Consequently, surgical success should not be assessed solely through radiological parameters; it must also incorporate functional outcomes and patient-reported quality of life measures [2].

The purpose of this research is to evaluate and compare postoperative foot health and pain outcomes in patients who have undergone Chevron or SERI procedures for hallux valgus. By employing the Foot Health Status Questionnaire (FHSQ) and pain scoring tools, the study aims to determine which technique provides superior functional recovery and quality of life, thus informing evidence-based clinical decisions.

## Materials and methods

This cross-sectional study was conducted in the Orthopedic Department of Security Forces Hospital in Riyadh, Saudi Arabia, within a timeframe from September 2022 to September 2024. The study sought to assess and compare patient reported postoperative outcomes of two surgical approaches for the treatment of isolated hallux valgus: the SERI and Chevron procedures. The study sample comprised patients of both sexes and all age demographics who underwent one of the two surgeries specifically for isolated hallux valgus.

Patients were excluded if they had additional concurrent forefoot abnormalities such as hammer toe or diabetic foot, had another concomitant surgery at the same time besides SERI or Chevron procedures, had recurrent hallux valgus, possessed insufficient hospital record documentation, or were unreachable despite numerous attempts. All patient was confirmed to be managed with full cast during the immediate postoperative period for a range of five to six weeks with no orthotics was prescribed (e.g., plantar, Morton's pads, or spacers) as standard postoperative protocol in the institution. Patients were followed up two, six, 24, 48 weeks, and four years postoperatively.

After obtaining Institutional Review Board (IRB) approval number 24-780-77. The data were retrospectively acquired from the hospital's TrakCare electronic medical records system (InterSystems Corporation, Cambridge, MA) and transferred into an Excel spreadsheet (Microsoft Corp., Redmond, WA). The gathered information comprised each patient's file number, age, gender, diagnostic details, and telephone number. Both the operative interventions were conducted by two consultant surgeons who shared a comparable academic foundation and specialization in orthopedic foot and ankle surgery. The principal outcome measures were derived from the FHSQ, evaluating four essential domains at a period of two to four years postoperatively: foot pain, foot function, footwear comfort, and general foot health. The questionnaire comprised four sections: demographic data, comorbidities, visual analogue scale (VAS) score, and FHSQ score components. It was disseminated electronically with a Google Form (Google LLC, Mountain View, CA) link transmitted via WhatsApp (Meta Platforms, Inc., Menlo Park, CA) to the patients. In instances when patients could not access or utilize the electronic format, a direct phone contact was conducted to administer the survey verbally. Informed consent was obtained electronically prior to participation. The first page of the online questionnaire presented the consent form, which participants were required to acknowledge before proceeding to the survey questions.

Participants were categorized by surgical technique (SERI vs. Chevron). FHSQ scores (continuous variables) were compared between groups. Hallux valgus severity was classified preoperatively by intermetatarsal angle (IMA: mild <11°, moderate 11-16°, severe >16°) to analyze subgroup outcomes [11,12]. The utilization of a validated and widely recognized instrument like the FHSQ [13,14] mitigated measurement bias; however, potential biases included response bias from self-reporting, selection bias from non-responders, and recall bias, as patients reported outcomes from up to 12 months post-surgery. These were alleviated, in part, by providing various methods for response collection and ensuring that all eligible patients were reached.

To accurately calculate the FHSQ scores, the data were input into the FHSQ Data Analysis Software (version 1.04)(University of Sydney, Sydney, Australia) and analyzed using SPSS version 26.0 (IBM Corp., Armonk, NY). Quantitative variables were summarized using mean, standard deviation, median, and interquartile range (IQR), while categorical variables were presented as frequencies and percentages. The primary analysis focused on comparing treatment groups at all time points for both primary and secondary outcomes. Data distribution was evaluated using Kolmogorov-Smirnov tests. For normally distributed data at a single time point, independent t-tests were applied, whereas mixed-model ANOVA with post hoc comparisons was used for multiple time points. Non-normally distributed data at one time point were analyzed using Mann-Whitney U or Kruskal-Wallis tests, depending on the number of groups. Effect sizes were estimated using Cohen’s d to assess the magnitude of differences. Statistical significance was set at p-value < 0.05, with 95% confidence intervals (CIs) indicating result precision. To ensure data completeness, the online questionnaire was designed with mandatory fields, requiring a response to each item to allow submission. An optional "prefer not to answer" choice was provided for all questions. Participants who selected this option (which occurred in only three instances across two patients) were excluded from the final analysis using listwise deletion.

## Results

A total of 58 patients were deemed eligible and invited to participate in the study; however, only 41 patients completed the questionnaire and were subsequently included in the analysis, representing a 70.7% response rate.

The SERI group included 24 patients, with four being males, while the Chevron group included 17 patients, with no males among the group. The mean age of the total sample was 32.5 years (±1.81), with patients who underwent Chevron osteotomy being older, 35.1 years (±3.43), with no statistically significant difference (p-value = 0.288). Subject characteristics, as well as footwear characteristics, are shown in Table 1. Comorbidities are shown in Table 2.

**Table 1:** Demographic characteristics of study population Statistical significance was defined as p < 0.05 (with a 95% confidence interval) in all analyses. SD = standard deviation; SERI = Simple, Effective, Rapid, and Inexpensive technique

Question	SERI osteotomy (N = 24)	Chevron osteotomy (N = 17)	Total (N = 41)
Age (years)			
Mean (±SD)	30.6 (±1.9)	35.1 (±3.4)	32.5 (±1.8)
Gender, n (%)			
Male	4 (16.7)	0 (0.0)	4 (9.8)
Female	20 (83.3)	17 (100)	37 (90.2)
Marital status			
Married	8 (34.8)	5 (33.3)	13 (34.2)
Single	15 (62.2)	10 (66.7)	25 (65.8)
Educational level			
High school or lesser	4 (16.7)	5 (29.4)	9 (22.0)
Diploma	6 (25.0)	2 (11.8)	8 (19.5)
University or higher	14 (58.3)	10 (58.8)	14 (58.5)
Occupational status			
Unemployed	7 (29.2)	6 (35.3)	13 (31.7)
Employed with no prolonged standing (<50% of a shift time)	11 (45.8)	8 (47.1)	19 (46.3)
Employed with prolonged standing (>50% of a shift time)	6 (25.0)	3 (17.6)	9 (22.0)
Monthly income (USD)			
Low income (<1800)	10 (50.0)	7 (46.7)	17 (48.6)
Middle income (1800-4000)	10 (50.0)	8 (53.3)	18 (51.4)
High income (>4000)	0 (0.0)	0 (0.0)	0 (0.0)
Exercise (hours a week)			
Not exercising at all	10 (41.7)	14 (82.4)	24 (58.5)
Exercising, less than 2.5	4 (16.7)	2 (11.8)	6 (14.6)
Exercising, more than 2.5	10 (41.7)	1 (5.9)	11 (26.8
Footwear type			
Walking shoe	16 (66.7)	11 (64.7)	27 (65.9)
Boot - UGG boot	0 (0.0)	0 (0.0)	0 (0.0)
Slipper	4 (16.7)	4 (23.5)	8 (19.5)
Backless slipper	3 (12.5)	2 (11.8)	5 (12.2)
Moccassins	1 (4.2)	0 (0.0)	1 (2.4)
Others	0 (0.0)	0 (0.0)	0 (0.0)
Type of footwear sole material			
Rubber	12 (66.7)	11 (68.8)	23 (67.7)
Plastic	3 (16.7)	3 (18.8)	6 (17.6)
Leather	3 (16.7)	2 (12.5)	5 (14.7
Wood	0 (0.0)	0 (0.0)	0 (0.0)

**Table 2:** Prevalence of comorbidities among males and females Percentages are representative of the total sample size (N = 41). Statistical significance was defined as p < 0.05 (with a 95% confidence interval) in all analyses.

Comorbidity, n (%)	SERI osteotomy (N = 24)	Chevron osteotomy (N = 17)
Diabetes mellitus	0 (0.0)	3 (7.3)
Hypertension	3 (7.3)	1 (2.4)
Thyroid diseases	0 (0.0)	1 (2.4)
Asthma	2 (8.3)	0 (0.0)

The analysis in Table 3 revealed significant differences between SERI and Chevron osteotomy in foot function and GFH domains. Patients undergoing SERI osteotomy reported superior foot function (mean = 73.96, 95% CI: 64.19-83.73) compared to Chevron (mean = 53.56, 95% CI: 38.70-68.41), with a statistically significant difference (p-value = 0.023) and a moderate effect size (Cohen’s d = 0.795). Similarly, the SERI group demonstrated markedly better GFH scores (mean = 78.33, 95% CI: 69.94-86.73) versus Chevron (mean = 57.21, 95% CI: 43.94-70.47), with a stronger effect (p-value = 0.008; Cohen’s d = 0.939).

**Table 3:** The impact of SERI versus Chevron osteotomy on foot-specific health-related quality of life Measured using the FHSQ scores. Statistical significance was defined as p < 0.05 (with a 95% confidence interval) in all analyses. CI = confidence interval; FHSQ = Foot Health Status Questionnaire; GFH = general foot health; SERI = Simple, Effective, Rapid, and Inexpensive technique; VAS = visual analogue score

FHSQ domain	Total group mean (CI), N = 41	SERI osteotomy mean ± SD (range), N = 24	Chevron osteotomy mean ± SD (range), N = 17	p-value	Effect size (Cohen’s d)
Foot pain	59.98 (51.85, 68.11)	63.88 (53.38, 74.39)	54.47 (40.75, 68,20)	0.327	0.367 (weak)
Foot function	65.50 (56.89, 74.12)	73.96 (64.19, 83.73)	53.56 (38.70, 68.41)	0.023*	0.795 (moderate)
Footwear	39.13 (30.43, 47.81)	41.32 (30.12, 52.52)	36.03 (21.00, 51.16)	0.592	0.191 (weak)
GFH	69.57 (61.81, 77.34)	78.33 (69.94, 86.73)	57.21 (43.94, 70.47)	0.008*	0.939 (high)
VAS score					
0-100 mm score	35.01 (25.68, 45.34)	30.00 (16.72, 43.28)	41.00 (24.28, 59.52)	0.234	0.038 (weak)

For other domains, differences were non-significant. Foot pain (SERI: 63.88 vs. Chevron: 54.47; p-value = 0.327, d = 0.367) and footwear (SERI: 41.32 vs. Chevron: 36.03; p-value = 0.592, d = 0.191) showed weak effects, suggesting comparable outcomes. The Chevron group had higher but non-significant VAS pain scores (41.00 vs. 30.00; p-value = 0.234, d = 0.038).

The analysis of postoperative outcomes based on preoperative severity levels revealed notable differences between the SERI and Chevron osteotomy groups. In the foot function domain, patients with moderate pre-operative severity who underwent the SERI procedure reported significantly better outcomes (mean = 78.3, CI: 67.2 to 89.4, p-value = 0.029) compared to the Chevron group (mean = 55.4, CI: 32.6 to 77.2). Similarly, in the GFH domain, the SERI group demonstrated superior results for moderate cases (mean = 72.0, CI: 61.8 to 82.1, p-value = 0.016) versus the Chevron group (mean = 45.5, CI: 27.6 to 63.4) (Table 4).

**Table 4:** Comparison between SERI and Chevron osteotomy among different pre-operative severity levels for self-reported FHSQ scores Statistical significance was defined as p < 0.05 (with a 95% confidence interval) in all analyses. CI = confidence interval; FHSQ = Foot Health Status Questionnaire; SERI = Simple, Effective, Rapid, and Inexpensive technique

FHSQ domain	Severity	Total group mean (CI), N = 41	SERI osteotomy mean (CI), N = 24	Chevron osteotomy mean (CI), N = 17	Mann-Whitney U
Foot pain domain					
-	Mild	51.0 (-3.0, 105.1)	34.5 (-323.0, 391.7)	67.7 (-180.9, 316.3)	0.439
-	Moderate	58.8 (48.1, 69.5)	64.2 (52.9, 75.6)	51.02 (26.95,75.08)	0.289
-	Severe	67.6 (56.2, 78.9)	74.4 (52.5, 96.3)	54.64 (29.60, 79.69)	0.292
Within group p-value		0.541	0.348	0.550	-
Foot function domain					
-	Mild	47.9 (0.6, 95.3)	43.8 (-35.7, 123.2)	52.1 (-397.9 , 502.1)	1
-	Moderate	71.2 (60.3, 82.1)	78.3 (67.2, 89.4)	55.4 (32.6, 77.2)	0.029
-	Severe	55.6 (41.1, 70.1)	59.4 (29.5, 89.2)	52.5 (27.0, 78.0)	0.700
Within group p-value	-	0.081	0.077	0.957	-
Footwear domain					
-	Mild	39.8 (-22.4, 102.0)	12.5 (-146.3, 171.3)	67.1 (-255.9, 390.0)	0.121
-	Moderate	37.9 (27.1, 48.8)	37.7 (24.4, 51.0)	34.5 (11.9, 57.1)	0.339
-	Severe	42.6 (24.9, 60.3)	62.5 (49.2, 75.8)	26.7 (5.5, 47.9)	0.054
Within group p-value	-	0.829	0.116	0.254	-
General foot health domain					
-	Mild	86.3 (56.2, 116.3)	100.0 (100.0, 100.0)	72.5 (-86.3, 231.3)	0.102
-	Moderate	63.6 (53.6, 73.6)	72.0 (61.8, 82.1)	45.5 (27.6, 63.4)	0.016
-	Severe	80.8 (68.8, 92.9)	88.8 (76.8, 100.7)	75.6 (51.7, 97.4)	0.080
Within group p-value	-	0.075	0..072	0.084	-

For other domains and severity levels, the differences between the two procedures were less pronounced. In the foot pain domain, no statistically significant differences were observed between the SERI and Chevron groups across mild, moderate, or severe cases (p-values > 0.05). The footwear domain also showed no significant differences, though the SERI group exhibited a trend toward better outcomes in severe cases (mean = 62.5, CI: 49.2 to 75.8, p-value = 0.054).

## Discussion

The primary goal of hallux valgus surgery is pain relief. Since pain and functional impairment are subjective, patient-reported outcome measures (PROMs) are essential for accurately assessing treatment success, as they directly reflect patient-perceived improvement [2]. While radiological and clinical parameters are commonly used to evaluate outcomes, they may not fully align with patient satisfaction, emphasizing the importance of PROMs in determining a procedure’s efficacy.

For mild-to-moderate hallux valgus, the Chevron osteotomy remains a widely used open technique, with outcomes comparable to the Scarf osteotomy [15,16]. In contrast, the SERI technique is a newer, minimally invasive approach gaining popularity. Both methods are well-documented as safe and effective [16].

This study assessed foot-specific quality of life and pain using the FHSQ and VAS in 41 patients undergoing Chevron or SERI osteotomy, stratified by hallux valgus severity (based on IMA). While multiple studies compare Chevron to other MISs, few specifically examine SERI [6,17]. Prior research indicates that MIS approaches, including SERI, offer early postoperative advantages, with comparable long-term clinical and radiographic outcomes to Chevron [9,18]. However, only one study directly compared Chevron and SERI, finding similar radiological outcomes and complications incidence at one year but better MTPJ mobility and lower recurrence rates (4.3% vs. 17.1%) with SERI [6,8]. Notably, neither study evaluated passive range of movement, leaving a critical gap in assessing patient-centered outcomes.

Our findings revealed that SERI yielded significantly better FHSQ scores in moderate hallux valgus cases, likely due to its minimally invasive nature, which reduces surgical trauma, scarring, and stiffness compared to open Chevron osteotomy [7,19]. A large-scale study of open versus MIS (n > 1,000 feet) confirmed these benefits, particularly in moderate deformities where adequate correction can be achieved while preserving MIS advantages [19,20].

Preoperative FHSQ scores from prior studies were lower than our postoperative results, possibly due to differences in hallux valgus severity classification [21]. Comparisons with the general population showed that post-surgical GFH scores aligned with healthy controls, while other domains remained lower, particularly in females, those with lower education, income, or physical activity levels [22,23].

Although SERI patients reported slightly lower pain scores (VAS), the difference was not statistically significant, consistent with literature findings [24]. A meta-analysis noted early postoperative pain reduction with MIS techniques, likely due to less tissue disruption, but no long-term differences in pain scores [20]. Similarly, the only RCT on this topic found no significant differences in pain outcomes between techniques at any follow-up [18].

This study has several limitations that warrant consideration and temper the generalizability of our findings. First, the cross-sectional design precludes the establishment of causal relationships. Second, the modest sample size, particularly within the severity-stratified subgroups, limits the statistical power and increases the vulnerability of the results to type II errors. A notable demographic constraint is the significant gender imbalance within our cohort, which may confound the outcomes and limit the extrapolation of findings, particularly to male populations. Furthermore, while the FHSQ is a validated instrument, the subjective nature of PROMs introduces potential for recall bias, an issue exacerbated by the two- to four-year postoperative interval. Finally, the retrospective data collection and online survey distribution may have introduced selection and response biases, potentially favoring more digitally literate or satisfied patients. Future investigations should employ prospective, longitudinal designs with larger, multi-center cohorts that are more demographically representative to enhance statistical power and external validity. While our findings support the use of the SERI technique for moderate deformities, these results should be confirmed in larger, prospective studies before they can be formally integrated into surgical practice guidelines.

## Conclusions

Both Chevron and SERI osteotomies effectively improve pain and function in hallux valgus. SERI offers better patient-reported outcomes in moderate cases due to its minimally invasive approach, although long-term pain relief is similar. PROMs are essential for assessing success. Future studies should refine patient selection, but both techniques remain safe and reliable options.

## References

[1] Cai Y, Song Y, He M, He W, Zhong X, Wen H, Wei Q (2023). Global prevalence and incidence of hallux valgus: a systematic review and meta-analysis. J Foot Ankle Res.

[2] Kakwani M, Kakwani R (2021). Current concepts review of hallux valgus. J Arthroscopy Joint Sur.

[3] Keenan AM, Drake C, Conaghan PG, Tennant A (2019). The prevalence and impact of self-reported foot and ankle pain in the over 55 age group: a secondary data analysis from a large community sample. J Foot Ankle Res.

[4] Chang C, Wang QF, Guo JC, Li DD, Fan YB, Wen JM (2020). The biomechanical relationship between hallux valgus deformity and metatarsal pain. J Healthc Eng.

[5] Rosemberg DL, Gustafson JA, Bordignon G, Bohl DD, Leporace G, Metsavaht L (2023). Biokinetic evaluation of hallux valgus during gait: a systematic review. Foot Ankle Int.

[6] Palmanovich E, Ohana N, David S (2021). Distal chevron osteotomy vs the simple, effective, rapid, inexpensive technique (SERI) for mild to moderate isolated hallux valgus: a randomized controlled study. Indian J Orthop.

[7] Kuliński P, Rutkowski M, Tomczyk Ł, Miękisiak G, Morasiewicz P (2023). Outcomes after chevron osteotomy with and without additional Akin osteotomy: a retrospective comparative study. Indian J Orthop.

[8] Almalki T, Alatassi R, Alajlan A, Alghamdi K, Abdulaal A (2019). Assessment of the efficacy of SERI osteotomy for hallux valgus correction. J Orthop Surg Res.

[9] Giannini S, Faldini C, Nanni M, Di Martino A, Luciani D, Vannini F (2013). A minimally invasive technique for surgical treatment of hallux valgus: simple, effective, rapid, inexpensive (SERI). Int Orthop.

[10] Saro C, Jensen I, Lindgren U, Felländer-Tsai L (2007). Quality-of-life outcome after hallux valgus surgery. Qual Life Res.

[11] Ray JJ, Friedmann AJ, Hanselman AE (2019). Hallux valgus. Foot Ankle Orthop.

[12] Ortiz C, Wagner P, Vela O, Fischman D, Cavada G, Wagner E (2016). "Angle to be corrected" in preoperative evaluation for hallux valgus surgery: analysis of a new angular measurement. Foot Ankle Int.

[13] Spruce MC, Bowling FL, Metcalfe SA (2011). A longitudinal study of hallux valgus surgical outcomes using a validated patient centred outcome measure. Foot (Edinb).

[14] Alshammari S, Alshwieer MA, Dammas SS, Alrasheed AM, Alasmari MA, Alahmari MM, Alazmi AK (2023). Arabic translation, cross cultural adaptation, and validation of Foot Health Status Questionnaire among Saudi individuals with plantar fasciitis. J Orthop Surg Res.

[15] Matar HE, Platt SR (2021). Overview of randomised controlled trials in hallux valgus surgery (2,184 patients). Foot Ankle Surg.

[16] Elgoyoushi SM, Omar MA, Dabash S, Thabet AM, Elbatrawy Y (2020). Analysis of different osteotomies used in hallux valgus: a systematic review. J Limb Lengthen Reconstr.

[17] Trnka HJ (2021). Percutaneous, MIS and open hallux valgus surgery. EFORT Open Rev.

[18] Kaufmann G, Dammerer D, Heyenbrock F, Braito M, Moertlbauer L, Liebensteiner M (2019). Minimally invasive versus open chevron osteotomy for hallux valgus correction: a randomized controlled trial. Int Orthop.

[19] Brogan K, Lindisfarne E, Akehurst H, Farook U, Shrier W, Palmer S (2016). inimally invasive and open distal chevron osteotomy for mild to moderate hallux valgus. Foot Ankle Int.

[20] Ji L, Wang K, Ding S, Sun C, Sun S, Zhang M (2022). Minimally invasive vs. open surgery for hallux valgus: a meta-analysis. Front Surg.

[21] Nix SE, Vicenzino BT, Smith MD (2012). Foot pain and functional limitation in healthy adults with hallux valgus: a cross-sectional study. BMC Musculoskelet Disord.

[22] Almaawi A, Alqarni H, Thallaj AK, Alhuqbani M, Aldosari Z, Aldosari O, Alsaber N (2023). Foot health and quality of life among adults in Riyadh, Saudi Arabia: a cross-sectional study. J Orthop Surg Res.

[23] Alshammari SA, Alshwieer MA, Dammas SS, Alrasheed AM, Alasmari MA, Alahmari MM (2023). Impact of plantar fasciitis on foot-specific and generic health-related quality of life in King Khalid University Hospital, Saudi Arabia. Cureus.

[24] Hernández-Castillejo LE, Martínez Vizcaíno V, Garrido-Miguel M, Cavero-Redondo I, Pozuelo-Carrascosa DP, Álvarez-Bueno C (2020). Effectiveness of hallux valgus surgery on patient quality of life: a systematic review and meta-analysis. Acta Orthop.

